# 3D Amide Proton Transfer Weighted Brain Tumor Imaging With Compressed SENSE: Effects of Different Acceleration Factors

**DOI:** 10.3389/fnins.2022.876587

**Published:** 2022-05-26

**Authors:** Nan Zhang, Haonan Zhang, Bingbing Gao, Yanwei Miao, Ailian Liu, Qingwei Song, Liangjie Lin, Jiazheng Wang

**Affiliations:** ^1^Department of Radiology, First Affiliated Hospital of Dalian Medical University, Dalian, China; ^2^Department of Radiology, Zhongshan Hospital of Fudan University, Shanghai, China; ^3^MSC Clinical and Technical Solutions, Philips Healthcare, Beijing, China

**Keywords:** amide proton transfer, brain tumor, compressed SENSE, acceleration factor, peritumoral edema

## Abstract

**Objectives:**

The aim of the current study was to evaluate the performance of compressed SENSE (CS) for 3D amide proton transfer weighted (APTw) brain tumor imaging with different acceleration factors (AFs), and the results were compared with those of conventional SENSE.

**Methods:**

Approximately 51 patients with brain tumor (22 males, 49.95 ± 10.52 years) with meningiomas (*n* = 16), metastases (*n* = 12), or gliomas (*n* = 23) were enrolled. All the patients received 3D APTw imaging scans on a 3.0 T scanner with acceleration by CS (AFs: CS2, CS3, CS4, and CS5) and SENSE (AF: S1.6). Two readers independently and subjectively evaluated the APTw images relative to image quality and measured confidence concerning image blur, distortion, motion, and ghosting artifacts, lesion recognition, and contour delineation with a 5-point Likert scale. Mean amide proton transfer (APT) values of brain tumors (APT_*tumor*_), the contralateral normal-appearing white matter (APT_*CNAWM*_), and the peritumoral edema area (if present, APT_*edema*_) and the tumor volume (V_*APT*_) were measured for objective evaluation and determination of the optimal AF. The Ki67 labeling index was also measured by using standard immunohistochemical staining procedures in samples from patients with gliomas, and the correlation between tumor APT values and the Ki67 index was analyzed.

**Results:**

The image quality of AF = CS5 was significantly lower than that of other groups. V_*APT*_ showed significant differences among the six sequences in meningiomas (*p* = 0.048) and gliomas (*p* = 0.023). The pairwise comparison showed that the V_*APT*_ values of meningiomas measured from images by CS5 were significantly lower, and gliomas were significantly larger than those by SENSE1.6 and other CS accelerations, (*p* < 0.05). APT_*tumor*_ (*p* = 0.191) showed no significant difference among the three types of tumors. The APT_*tumor*_ values of gliomas measured by APTw images with the SENSE factor of 1.6 and the CS factor of 2, 3, and 4 (except for CS5) were all positively correlated with Ki67.

**Conclusion:**

Compressed SENSE could be successfully extended to accelerated 3D APTw imaging of brain tumors without compromising image quality using the AF of 4.

## Introduction

Although head magnetic resonance imaging (MRI) is an established tool for head tumor diagnosis in clinical practice, standard MRI sequences (such as T2 weighted imaging, T2W; fluid-attenuated inversion recovery, FLAIR; diffusion-weighted imaging, DWI; and gadolinium-enhanced T1-weighted imaging, Gd-T1W) can provide plentiful information for detection and differentiation of brain tumors. However, the diagnostic specificity is still limited in differentiating grades of brain tumors and predicting proliferation potential. When diagnosing malignant gliomas, T2W hyperintensity may represent both peritumoral vasogenic edema and infiltrating tumor. Gd-T1W also could not reliably distinguish the lesions of necrotic and highly cell-infiltrating tumors (Eidel et al., 2017). Besides, different kinds of perfusion, diffusion and MR spectroscopic imaging (MRSI) methods have been introduced to clinical or research protocols, but their stability and accuracy are still controversial.

Chemical exchange saturation transfer (CEST) imaging is a novel magnetic resonance molecular imaging method derived from magnetization transfer (MT) technology (van Zijl et al., 2018). Selective pre-saturation is carried out by applying saturation radio-frequency (RF) pulses to endogenous or exogenous specific substances, and the interexchange between saturated hydrogen protons and hydrogen protons in free water molecules causes the attenuation of free water signals (van Zijl and Yadav, 2011; Zhou et al., 2019). The degree of water signal attenuation is proportional to the number of targeted compounds with exchangeable protons; therefore, CEST enables indirect measurement of tissue metabolites, as well as a reflection of the physical and chemical properties of tissues that affect the chemical exchange (van Zijl and Yadav, 2011; Pankowska et al., 2019). Amide proton transfer-weighted (APTw) MR imaging, as a kind of CEST technology targeting amide protons (resonating at 3.5 ppm downfield from water), has provided a tool for non-invasive detection of endogenous free proteins and polypeptide molecules in the cytoplasm, and hence, the indirect measurement of metabolic changes and pathophysiological information in living cells (Zhou et al., 2013; Lin et al., 2018; Ishimatsu et al., 2019; Debnath et al., 2020). APTw MRI was introduced in 2003 for imaging brain tumors (Zhou et al., 2003). Currently, the most common application of APTw MRI is for the study of human brain tumors (Wen et al., 2010; Jiang et al., 2016; Yu et al., 2017; Joo et al., 2018), where it starts to impact grade (Xu et al., 2021), recurrence (Park et al., 2020), and chemotherapy response (Joo et al., 2018) of glioma, differentiation of meningiomas (Scott et al., 2002) and brain metastases (Yu et al., 2017). Numerous studies from different institutions (Scott et al., 2002; Park et al., 2021; Xu et al., 2021) indicate that APTw imaging may add important value to brain cancer diagnosis. The added value of APTw MRI in enhancing the non-invasive diagnosis of brain tumors at a molecular level can potentially aid in guiding brain tumor therapies, such as surgery, radiotherapy, and local chemotherapy.

However, CEST imaging, including APTw MRI, can be much slower than conventional qualitative imaging because the saturated image needs to be repeated at multiple saturation frequencies, especially when the three-dimensional acquisition is required for whole-brain coverage (Zhu et al., 2010). Even longer scan times are required to obtain a higher spectral resolution Z spectrum with a wide bias frequency range (Zhang et al., 2020). The extensive clinical applications of APTw MRI require rapid volume imaging. Volume (3D) acquisition is more inclined to minimize the difference in saturation loss caused by T1 relaxation between slices (Zhu et al., 2010). Zhu et al. (2010) developed a whole-brain 3D APTw MRI method with a higher spatial resolution that had great advantages, especially for describing intra-tumor heterogeneity. But it is subject to a long scanning time. Patients with brain tumors are poorly tolerated and can be predisposing to a motion artifact and limiting the ability to incorporate such pulse sequences into clinical imaging time slots. APTw imaging is very sensitive to movement. Therefore, shortening the scanning time is very necessary for APTw imaging.

Therefore, scan time is one of the key factors that limit the application of CEST-MRI in clinical use. Compressed sensing (CS; Candes et al., 2006; Donoho, 2006) is a new framework for data acquisition and signal recovery. It allows measuring sparse and compressible signals at a rate close to their intrinsic information rate rather than their Nyquist rate. These signals can be reconstructed exactly from very few incoherent measurements by a non-linear procedure (Donoho, 2006; Candès et al., 2007). CS aims to reconstruct signals and images from significantly fewer measurements than were traditionally thought necessary. Magnetic Resonance Imaging is an essential medical imaging tool burdened by an inherently slow data acquisition process. The application of CS to MRI has the potential for significant scan time reductions, with benefits for patients and health care economics. Previous studies (Togao et al., 2017; Heo et al., 2019) have demonstrated the feasibility of compressed sensing (CS) for accelerated CEST-MRI in healthy volunteers and patients with brain tumors with an acceleration factor (AF) of 4 (Gawlitza et al., 2017), but whether different accelerators have effects on APTw imaging remains to be further explored. The current study aimed to explore the performance of the compressed SENSE (CS-SENSE) technique (Heo et al., 2019) for the acceleration of brain tumor APTw imaging using different AFs and to find the optimal AF.

## Materials and Methods

### Patients

**FIGURE 1:**
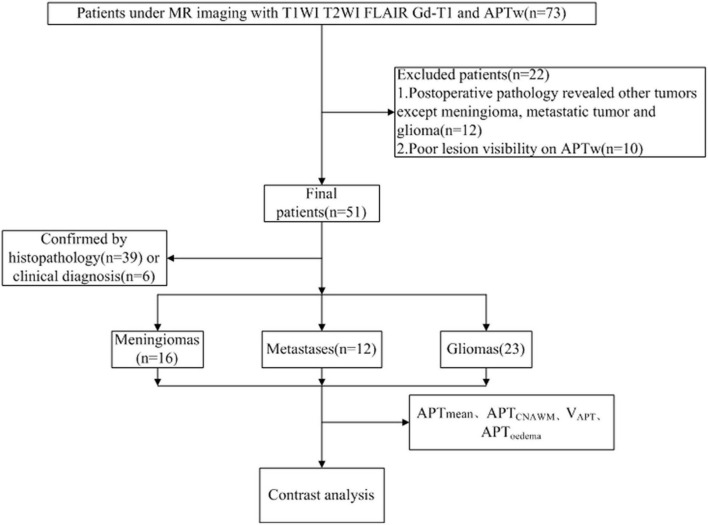
A flow diagram of the patient selection process.

The study was approved by the Local Ethics Committee, and all the patients were informed and gave their written consent to participate in this study. Between October 2019 and March 2021, a total of 73 patients were included with the criteria of (1) previously untreated; (2) underwent a complete multiparametric MRI exam consisting of APTw and Gd-enhanced T1W imaging; and (3) histopathological confirmation was obtained by gross total or partial surgical resection and by stereotactic biopsy after the multiparametric MRI exam. Approximately, 22 patients were excluded from the study for the following reasons: 1. Postoperative pathology revealed other tumors except for meningioma, metastatic tumor, and glioma (12 cases); 2. Poor lesion visibility caused by patient movement on APTw images (10 cases). Finally, 51 patients [22 males, age: 49.95 ± 10.52 (24–68) years] were included in the study, who were divided into three groups (Figure 1): 1. Meningiomas (*n* = 16), including 5 pathologically diagnosed with atypical meningioma, and 11 with benign meningioma; 2. Metastases (*n* = 12), the primary sites of cancer included lung (*n* = 6), breast (*n* = 2), kidney (*n* = 1), colon (*n* = 1), and liver (*n* = 2); 3. Gliomas (*n* = 23), including Grade II(*n* = 6), Grade III(*n* = 7), Grade IV(*n* = 10).

### Surgery and Pathologic Evaluation

Histopathological confirmation was obtained by gross total or partial surgical resection and by stereotactic biopsy. Tumor types were determined based on the pathological diagnosis of the surgical specimens according to the 2016 World Health Organization (WHO) classification. Gliomas were classified with Grades II, III, and IV. The Ki67 labeling index was also measured by using standard immunohistochemical staining procedures in samples from patients with gliomas. Tumor sections were reviewed and Ki67 was quantified based on the percentage of positive cells in the highest density of the stained areas; all cells with nuclear staining of any intensity were considered positive, and the Ki67 labeling index was defined as the percentage of positive cells among the total number of counted cells.

### Magnetic Resonance Imaging Protocol

Magnetic resonance examinations were performed on a 3.0 T MR scanner (Ingenia CX, Philips Healthcare, Netherlands) with a 32-channel head coil. Each patient was placed in a supine position without compression to the head. The APTw images were acquired in addition to the conventional imaging protocols (T1W, T2W, FLAIR, and Gd-T1W), with imaging parameters detailed in Table 1. The CS acceleration factors used in this study included 2, 3, 4, and 5. For APTw imaging, a 2-s saturation pulse was applied with 2-μT B1 amplitude at each of the following 6 frequencies for a Z spectrum: ±2.7 ppm, ±3.5 ppm, and ±4.3 ppm, where 0 ppm was defined as the water proton resonance. A reference acquisition was performed with the radio frequency (RF) at −1,560 ppm. Three acquisitions were made with the RF saturation at +3.5 ppm and with echo shifts to generate a B0 map for voxel-wise frequency correction to the Z spectra. To ensure acceptable spectral selectivity, we enforced a spectral resolution smaller than 100 Hz measured as the full width at half maximum by acquiring spectral signals before each APTw scan. The APTw sequences were scanned before the injection of the gadolinium contrast agent (Gadodiamide, Bayer AG, Bayer Leverkusen, Germany).

**TABLE 1:** Magnetic resonance (MR) parameters of all sequences of brain imaging.

	T1W	T2W	T2 FLAIR	Gd-T1W	APTw
Scan mode	TSE	TSE	TSE	TFE	TSE
Repetition time, TR (ms)	2095	4000	9000	260	7280
Echo time, TE (ms)	15	122	125	4.6	7.8
Field of view, FOV (mm^2^)	230 × 230	230 × 230	230 × 187	240 × 240	230 × 180 × 70
Acquisition voxel size (mm^3^)	0.8 × 0.88	0.6 × 0.6	0.75 × 1.04	0.65 × 0.8	1.8 × 1.8 × 7
Recon voxel size (mm^3^)	0.48 × 0.48	0.45 × 0.45	0.53 × 0.53	0.45 × 0.45	0.9 × 0.9 × 7
Flip angle (degree)	110	90	110	11	90
Slice thickness (mm)	6.5	6.5	6.5	0.96	7
Gap (mm)	1	1	1	−0.48	0
TSE/TFE factor	7	30	39	64	174
Acceleration mode/factor R	SENSE2	SENSE2	SENSE2.1	SENSE1.5	SENSE: 1.6 CS: 2, 3, 4, 5
Saturation B1 amplitude (μT)	–	–	–	–	2
Saturation duration (s)	–	–	–	–	2
Scan time (s)	88	96	81	44	219, 219, 166, 113, 59.3

### Image Analysis

Amide proton transfer weighted images were automatically reconstructed on the MR console after the data acquisition according to APTw imaging theory and a previously published algorithm (Togao et al., 2017) and transferred to the IntelliSpace Portal (ISP v7.0, Philips Healthcare, Netherlands) workstation for amide proton transfer (APT) value measurements. For each patient, the threshold extraction method for segmentation of the tumors as a whole was carried out according to the following steps independently by two neuroradiologists (both with 3 years of experience): 1. The 3D tumor structure was first automatically segmented on GD-T1W images using the MR segmentation software on ISP; 2. Manual adjustment was implemented for the tumor segmentation to ensure the inclusion of all tumor; 3. The threshold within the range of 0–1% to 5–8% was manually selected to remove structures of blood vessels and necrosis; 4. The segmentations were copied to APTw images for measurement of tumor volumes (V_*APT*_) and the mean APT values of tumors (APT_*tumor*_). The whole tumor volumes (ROIs) were distributed within the Gd-enhancing tumor area, as well as within the peritumoral brain zone based on the Gd-T1WI and T2WI co-registered with the APTw image (Zhang et al., 2016). Also, the ROIs (with a size about 50 mm^2^) were manually defined on the solid parts of tumors in the contralateral normal-appearing white matter (CNAWM) area to get APT_*CNAWM*_ values. The peritumoral edema (surrounding non-enhancing areas with T2-prolongation, if present) was manually segmented using threshold fitting on the T2W images; tumor and necrosis were removed manually according to GD-T1W images. The segmented regions were also copied to APTw images for measurement of APT_*edema*_ values.

### Quantitative Analysis

Two readers independently and subjectively evaluated the APTw images relative to image quality and measured confidence with respect to image blur, distortion, motion and ghosting artifacts, lesion recognition, and contour delineation with a 5-point Likert scale: 5, good image quality with tumor detectable and lesion contour delineated on APTw images without reference to contrast-enhanced MR images; 4, tumor lesion could be recognized on APTw images, but contour was not so well delineated, reference information on contrast-enhanced MR images; 3, tumor undetectable without reference to contrast-enhanced MR; 2, poor APTw image quality with obvious artifacts, although the tumor lesion was revealed on T2WI or DWI images; 1, no lesions were identified on any contrast-enhanced MR or APTw images.

### Statistical Analyses

All statistical analyses were performed using the statistical package SPSS 21.0. Intraclass correlation coefficient (ICC) was used to evaluate measurement consistency. An ICC greater than 0.75 was considered to indicate good agreement. For image quality assessment, interobserver agreement was evaluated by ICC. ICC values of less than 0.4, 0.41–0.75, and greater than 0.75 were considered to indicate positive but poor, fair, and good agreement, respectively. Friedman test was used to compare the difference in image quality score data and multiple comparisons were performed between each pair of different AFs with Bonferroni correction. Repeated ANOVA was used to test the APT measurement results by sequences with different SENSE/CS AFs. ANOVA was used to compare the APT_*tumor*_ values measured for different types of tumors. Pairwise comparisons were carried out using a Wilcoxon signed-rank test with the *p* values corrected by Bonferroni correction. Spearman’s correlation coefficient was used to analyze the correlation between APTw and Ki67 expressions. *P* < 0.05 was recognized as statistically significant.

## Results

### Qualitative Analysis

The subjective scores of the two observers for APTw images with different AFs were consistent (ICC value >0.75). The data by the senior observer were selected for analysis, and the image scores of different AFs were 4.9 ± 0.6, 4.3 ± 0.3, 4.1 ± 0.5, 4.0 ± 0.6, and 2.3 ± 0.4, respectively. There was a statistical difference in image scores among different AFs (*p* < 0.001). The image quality of AF = CS5 was significantly lower than those of other groups (Table 2).

**TABLE 2:** Pairwise comparison between different AF scan image quality evaluation groups (*p*-value).

	S1.6	CS2	CS3	CS4	CS5
S1.6	–	1.000	0.875	0.324	<0.001
CS2	1.000	–	0.822	0.214	<0.001
CS3	0.875	0.822	–	0.360	<0.001
CS4	0.324	0.214	0.360	–	<0.001
CS5	<0.001	<0.001	<0.001	<0.001	–

### Comparison of Quantitative Measurements From Amide Proton Transfer Weighted Images With Different Acceleration Factors

**FIGURE 2:**
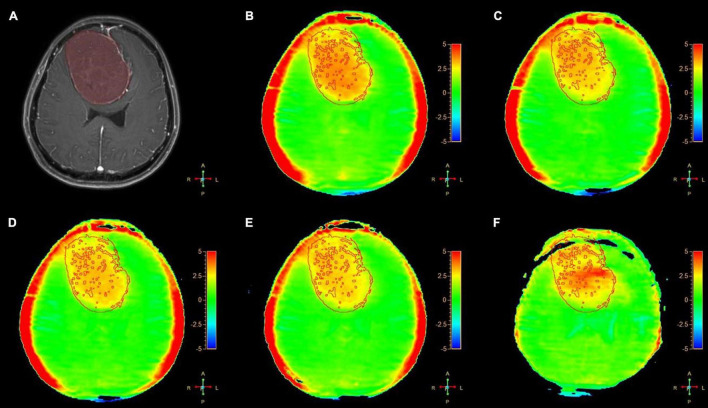
A 54-year-old female patient with meningiomas in brain: **(A)** Gd-T1W; **(B–F)** APTw images by SENSE (AF 1.6) and CS-SENSE (AFs of 2, 3, 4, and 5). The ROIs were obtained manually on the Gd-T1W image and copied to the others as shown.

**FIGURE 3:**
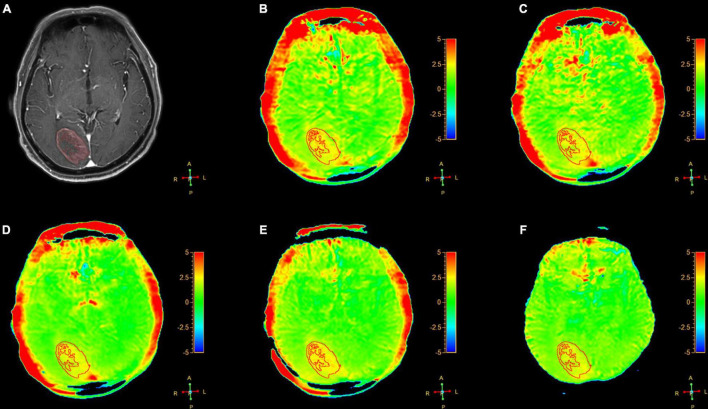
A 64-year-old female patient with metastasis in brain: **(A)** Gd-T1W; **(B–F)** APTw images by SENSE (AF 1.6) and CS-SENSE (AFs of 2, 3, 4, and 5). The ROIs were obtained manually on the Gd-T1W image and copied to the others as shown.

**FIGURE 4:**
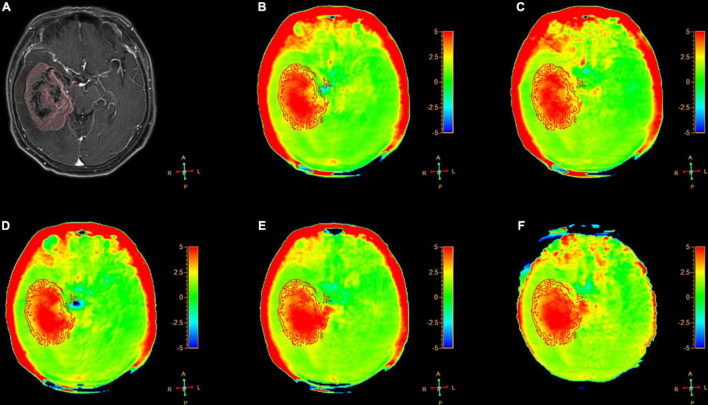
A 54-year-old female patient with gliomas tumors (Grade III) in brain: **(A)** Gd-T1W; **(B–F)** APTw images by SENSE (AF 1.6) and CS-SENSE (AFs of 2, 3, 4, and 5). The ROIs were obtained manually on the Gd-T1W image and copied to the others as shown.

**FIGURE 5:**
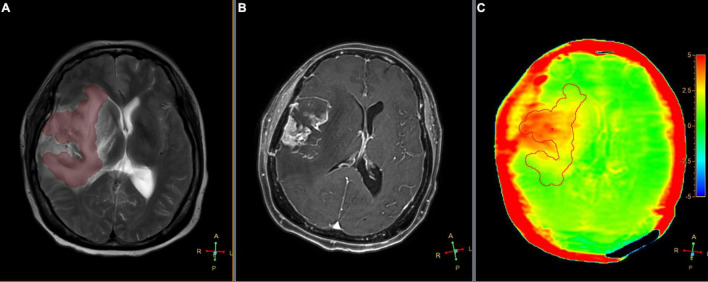
Segmented peritumoral high signal intensity areas and the peritumoral areas in a 63-year-old male with gliomas (Grade IV) overlaid onto coregistered T2W **(A)**, Gd-T1W **(B)**, and APTw **(C)** images (SENSE AF = 1.6). The APT_*edema*_ was 2.47%.

**FIGURE 6:**
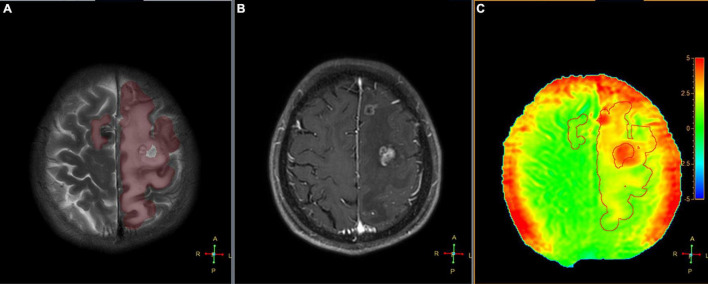
Segmented peritumoral high signal intensity areas and the peritumoral areas in a 45-year-old male with a metastasis (the primary site of cancer was lung) overlaid onto coregistered T2W **(A)**, Gd-T1W **(B)**, and APTw **(C)** images (SENSE AF = 1.6). The APT_*edema*_ was 2.54%.

Figures 2–6 showed the measurements of APT_*tumor*_, APT_*CNAWM*_ and V_*APT*_ values in the three types of tumors. APT_*CNAWM*_ and APT_*tumor*_ showed no significant difference among results by 3D APTw imaging with acceleration by CS (AFs = 2–5) and SENSE (AF = 1.6) in all the three types of tumors (*p* > 0.05), while the V_*APT*_ value showed significant differences among the six sequences in meningiomas (*p* = 0.048) and gliomas (*p* = 0.023) (Tables 3–5). The pairwise comparison showed that the V_*APT*_ values of meningiomas measured from images by CS5 were significantly lower than those by SENSE1.6 and other CS accelerations (*p* < 0.05) (Table 6), and the V_*APT*_ values of gliomas measured from images by CS5 were significantly larger than those by SENSE1.6 and other CS accelerations (*p* < 0.05) (Table 7).

**TABLE 3:** Comparisons of quantitative amide proton transfer (APT) values for meningiomas among CS and SENSE scans.

	APT_*CNAWM*_	APT_*tumor*_	V_*APT*_ (mm^3^)
SENSE1.6	0.81 ± 0.49	2.84 ± 0.74	10.03 ± 6.50
CS2	1.15 ± 0.51	3.08 ± 0.52	9.53 ± 6.41
CS3	0.99 ± 0.36	2.76 ± 0.53	9.94 ± 6.09
CS4	1.10 ± 0.60	2.85 ± 0.46	10.04 ± 6.22
CS5	1.04 ± 0.46	2.68 ± 0.27	2.28 ± 1.16
*p*	0.500	0.482	0.048

**TABLE 4:** Comparisons of quantitative APT values for metastasis among CS-SENSE and SENSE scans.

	APT_*CNAWM*_ (%)	APT_*tumor*_ (%)	V_*APT*_ (mm^3^)
SENSE1.6	1.08 ± 0.6	3.02 ± 0.90	7.23 ± 4.02
CS2	0.96 ± 0.55	2.89 ± 0.81	7.04 ± 4.52
CS3	0.83 ± 0.53	3.26 ± 0.80	6.97 ± 4.56
CS4	0.95 ± 0.55	2.72 ± 0.70	6.87 ± 4.70
CS5	0.38 ± 1.22	2.88 ± 0.72	5.23 ± 1.12
*p*	0.375	0.882	0.442

**TABLE 5:** Comparisons of quantitative APT values for gliomas between CS-SENSE and SENSE scans.

	APT_*CNAWM*_ (%)	APT_*tumor*_ (%)	V_*APT*_ (mm^3^)
SENSE1.6	0.93 ± 0.61	2.96 ± 0.63	110.23 ± 66.23
CS2	0.85 ± 0.61	2.76 ± 0.59	110.19 ± 62.22
CS3	0.87 ± 0.58	2.96 ± 0.48	109.89 ± 54.12
CS4	1.08 ± 0.42	2.92 ± 0.45	109.89 ± 43.18
CS5	0.79 ± 0.48	2.75 ± 0.61	140.23 ± 66.23
*p*	0.247	0.853	0.023

**TABLE 6:** The pairwise comparison of V_*APT*_ of meningiomas (*p-*value).

	S1.6	CS2	CS3	CS4	CS5
S1.6	–	0.482	0.634	0.543	0.012
CS2	0.482	–	0.563	1.000	0.014
CS3	0.634	0.563	–	0.562	0.023
CS4	0.543	1.000	0.562	–	0.021
CS5	0.012	0.014	0.023	0.021	–

**TABLE 7:** The pairwise comparison of gliomas of V_*APT*_ (*p-*value).

	S1.6	CS2	CS3	CS4	CS5
S1.6	–	1.000	0.943	0.654	0.032
CS2	1.000	–	0.762	0.832	0.026
CS3	0.634	0.762	–	0.743	0.024
CS4	0.654	0.832	0.743	–	0.034
CS5	0.032	0.026	0.024	0.034	–

### Comparison of Image Features From Amide Proton Transfer Weighted Images With Different Acceleration Factors

We assessed the radiologic characteristic of the meningiomas, metastases, and gliomas, using conventional (T1W, T2W, FLAIR, and Gd-T1W) and APTw sequences. For the meningiomas (16 cases), 11 cases showed homogenous enhancement and 5 showed heterogeneous enhancement on the Gd-T1W images. The tumor cores showed hyperintensities (relative to the CNAWM) on the APTw images, and the hyperintense areas on APTw images were approximately equal to the lesions shown on the Gd-T1W (Figure 2).

The Gd-T1W and APTw images for a patient with metastasis are illustrated in Figure 3. The tumor cores showed hyperintensities (relative to the CNAWM) on the APTw images, and the hyperintense areas on APTw images were approximately equal to the lesions shown on the Gd-T1W image.

The Gd-T1W and APTw images for a patient with glioma are illustrated in Figure 4. The APTw images overall showed visually homogenous APTw hyperintensities (spatially compared to the CNAWM) in the region with Gd enhancement on the Gd-T1W images.

### Comparison of Amide Proton Transfer Values Measured by the Optimal Acceleration Factor Among the Three Types of Tumors

The optimal AF of CS-SENSE determined in this study for 3D APTw brain tumor imaging was 4, which will be discussed later. Figures 2–4 show the measurements of APT_*tumor*_, APT_*CNAWM*_, and V_*APT*_ in the three types of tumors on images by the optimal AF of CS-SENSE. APT_*tumor*_ was higher than APT_*CNAWM*_ in all three types of tumors (*p* = 0.024). APT_*CNAWM*_ (*p* = 0.205) and APT_*tumor*_ (*p* = 0.191) showed no significant difference among the three types of tumors.

### Histological Composition of Different Magnetic Resonance Imaging Classifications

Of the total 23 gliomas (Grade II: *n* = 6, Grade III: *n* = 7, and Grade IV: *n* = 10), the APT_*tumor*_ values measured by APTw images with the SENSE factor of 1.6 and the CS factor of 2, 3, and 4 (except for CS5) were all positively correlated with Ki67 (Table 8).

**TABLE 8:** Correlation between APT_*tumor*_ and Ki67.

AF	APT_*tumor*_ (%)	*r*	*p*
SENSE1.6	2.96 ± 0.63	0.573	0.016
CS2	2.76 ± 0.59	0.590	0.013
CS3	2.96 ± 0.48	0.610	0.009
CS4	2.92 ± 0.45	0.621	0.008
CS5	2.75 ± 0.61	0.396	0.116

## Discussion

In the current study, brain APTw imaging accelerated by CS-SENSE with increased acceleration factors (AFs 2–5) was compared with results by conventional SENSE acceleration with an AF of 1.6. According to our results, the image quality was significantly reduced when the AF was increased to CS5, where the quantitatively measured volume (V_*APT*_) of the tumor was also significantly changed. Therefore, the acceleration factor 4 for CS-SENSE was recommended without significantly compromising image quality, saving at least 48.86% scan time compared to the SENSE acceleration.

Heo et al. (2019) have tried to apply compressed sensing for the reconstruction of fast scanned 3D APTw images (15 slices, slice thickness = 4 mm, plane spatial resolution = 1.8 mm^2^ × 1.8 mm^2^, and scan duration = 130 s) on patients with glioma based on the conventional parallel under-sampling scheme (GRAPPA). In contrast, the compressed SENSE method used in this study adopts an acquisition scheme of variable-density random under-sampling, and the sensitivity information from multi-channel coils was also included for the CS image reconstruction (Candes et al., 2006). Moreover, we presented a more comprehensive evaluation of the application of the compressed SENSE technique for accelerated 3D APTw imaging (with different acceleration factors ranging from 2-to 5) on an extended cohort of patients with a brain tumor. Also, the compressed SENSE with an acceleration factor of 4 (113 s) was recommended for 3D APTw brain tumor imaging without compromising image quality.

In previous studies, the APT values were usually measured from ROIs drawn on one single slice (Gawlitza et al., 2017; Yu et al., 2017; Xu et al., 2021), which might not be representative of tumors associated with strong heterogeneity. For each patient in this study, the 3D enhanced regions were automatically segmented on 3D Gd-T1W images using threshold fitting, and the segmented ROIs were copied to 3D APTw images. Therefore, the APT values measured in this study were from a 3D volume of the whole tumor, which can be more representative, especially for tumors with inhomogeneity.

The image quality of AF = CS5 was significantly lower than that of other groups. The APTw imaging with CS5 showed that the tumor undetectable without a reference to conventional MR images, the APT image of the tumor part only had decreased edge sharpness and signal drift, and the APT value was not affected.

Tumor volume was also studied in this study; the V_*APT*_ values of meningioma measured on APTw images by CS5 were significantly smaller than those measured on APTw images by other accelerations, and V_*APT*_ values of gliomas were larger than others. Gliomas may have peritumoral edema and necrosis. The APT values in peritumoral edema were significantly higher for gliomas than for meningiomas, which may represent the higher tumor cell infiltration of gliomas. This finding is consistent with Jiang et al. (2016). The V_*APT*_ values measured for metastasis showed no difference among all sequences. This indicates that the high CS acceleration factor (AF = 5) would have a significant influence on the display of glioma tumor volume. Numerous studies indicated that (Scott et al., 2002) gadolinium-enhanced MRI was limited in differentiation between low- and high-grade gliomas that some high-grade gliomas (roughly 10% of glioblastomas and 30% of anaplastic astrocytoma) demonstrate no gadolinium enhancement. Some cases of tumors in this study were observed with a larger area of hyperintense regions on APTw images than on Gd-T1W images. It can be seen that the size of APT hyperintense regions is of great significance to the display of tumor information, so the influence of excessive AF on the display of tumor volume cannot be ignored.

The peritumoral edema of meningioma was well displayed on the APTw images of the present study. Peritumoral edema in meningiomas was dependent on the expression of AQP-4 (Gawlitza et al., 2017). Joo et al. (2018) showed that both the peritumor edema of typical meningioma and benign meningioma could be observed on APTw images. Yu et al. (2019) showed that the mean APT value of peritumoral edema was not associated with tumor grade, but the APT_*min*_ and APT_*max–min*_ values of peritumoral edema were significantly different between WHO Grade I and Grade II meningiomas. The larger hyperintense regions in APTw maps than in Gd-T1W images in the cases of malignancies were observed in the current study, which may be attributed to the enhanced APT signals of peritumoral edema in malignancies.

For the metastases, the APT signal showed a high signal on peritumoral edema. Our results showed that the APT_*tumor*_ values in the enhancing areas (3.32 ± 1.09%) were significantly higher than APT_*edema*_ values (1.73 ± 0.2%). Previous studies (Zhou et al., 2013) have found that, in solitary brain metastases, the mean APT values in both the enhancing and edema areas were significantly higher than those in normal-appearing white matter. This study further investigated the APTw signal of peritumoral edema in metastatic tumors with accelerated 3D APTw imaging. The heterogeneity of the APTw signal was related to the heterogeneity of metastatic tumors, indicating that APTw imaging could reflect the necrosis of the metastatic tumor.

For the gliomas, the APT signal showed the peritumoral edema well, and the APT_*mean*_ with different AFs of the enhancement area was significantly different from that of the APT_*edema*_. The previous study by Jiang et al. (2016) has demonstrated that APT values of the peritumoral edema were significantly lower for primary central nervous system lymphomas than for high-grade gliomas. APT values of the peritumoral edema can be valuable for the differentiation of glioma from other tumors. When compared to DCE images, hyperintensity lesions on APTw maps were with more heterogeneous features, and similar results have been observed in previous studies (Jiang et al., 2016) for central nervous system lymphomas and gliomas. APTw imaging would be a valuable MRI biomarker by which to diagnose brain cancer. The Ki67 is a nuclear protein associated with tumor cell proliferation and the expression of ribosomal RNA. With a relatively short half-life, Ki67 serves as a sensitive marker for the proliferation of tumor cells. It has been demonstrated that Ki67 is positively correlated with the malignant degree of glioma (Wolfesberger et al., 2010), yet the relationship between Ki67 and the prognosis of glioma remains largely unknown. The sample size of this study is small, and it is only preliminary to conclude that Ki67 is related to the proliferation of glioma cells. In this study, the relationship between APT value and the positive expression rate of Ki67 was analyzed and results showed that APT_*tumor*_ values of gliomas measured by APTw images with the SENSE factor of 1.6 and the CS factor of 2, 3, and 4 (except for CS5) were all positively correlated with Ki67 (*p* < 0.05).

There are some limitations or weaknesses in this study. The tumor types included in this study were limited, and the sample size for each type of tumor was limited, which may be the reason for the lack of difference in APT_*tumor*_ among the three types of tumors. Further studies on the three types of tumors should be carried out with larger subjects.

## Conclusion

The compressed SENSE with an acceleration factor of 4 was recommended for 3D APTw imaging without compromising image quality for brain tumor imaging, and saving at least 48.86% scan time compared to the conventional SENSE acceleration. The APTw imaging can obtain molecule-level information about brain tumors, and the acceleration by compressed SENSE would benefit its clinical applications.

## Data Availability Statement

The original contributions presented in the study are included in the article/supplementary material, further inquiries can be directed to the corresponding author.

## Ethics Statement

The studies involving human participants were reviewed and approved by the Ethics Committee of First Affiliated Hospital of Dalian Medical University. The patients/participants provided their written informed consent to participate in this study.

## Author Contributions

NZ and QS conceived the study. NZ and HZ participated in the sequence alignment and scanning. BG and JW collected the clinical materials and data. AL and YM processed and analyzed the data. NZ wrote the first draft of the manuscript. LL revised the manuscript. All authors commented on previous versions of the manuscript and read and approved the final manuscript.

## Conflict of Interest

LL and JW are employee of Philips Healthcare, China. The remaining authors declare that the research was conducted in the absence of any commercial or financial relationships that could be construed as a potential conflict of interest.

## Publisher’s Note

All claims expressed in this article are solely those of the authors and do not necessarily represent those of their affiliated organizations, or those of the publisher, the editors and the reviewers. Any product that may be evaluated in this article, or claim that may be made by its manufacturer, is not guaranteed or endorsed by the publisher.

## Abbreviations

CS/CS-SENSE: compressed SENSE
APTw: amide proton transfer weighted
AF: acceleration factors
APT_*tumor*_: mean APT value of brain tumors
APT_*CNAWM*_: mean APT value of the contralateral normal appearing white matter
APT_*edema*_: mean APT value of the peritumoral edema area
V_*APT*_: tumor volume.
